# Development and Educational Evaluation of a Radiation Visualization System in a Virtual Reality Training Module for Radiopharmaceutical Administration: Mixed Methods Study of Students’ Learning Experiences

**DOI:** 10.2196/104397

**Published:** 2026-09-30

**Authors:** Akihiro Kakimoto, Daisuke Fujise, Shota Watanabe, Yutaka Otaka, Sakura Minemoto, Ayaka Yasumatsu, Shin Hasegawa

**Affiliations:** 1 Department of Radiological Sciences, Faculty of Medical Science Technology, Morinomiya University of Medical Sciences, 1-26-16 Nankokita, Suminoe-ku, Osaka-shi, Osaka, Osaka, 559-8611, Japan, 81 6-6616-6911, 81 6-6615-8965; 2 Department of Biofunctional Imaging, Preeminent Medical Photonics Education and Research Center, Hamamatsu University School of Medicine, Hamamatsu-shi, Shizuoka, Japan; 3 IT Planning Section, Department of Information Technology and Management, National Institutes for Quantum Science and Technology, Chiba-shi, Chiba, Japan

**Keywords:** education, radiological technologist students, virtual reality, radiological protection, radiopharmaceutical administration

## Abstract

**Background:**

Our research group previously developed a virtual reality (VR) training module for radiopharmaceutical administration, focusing on procedural skills and patient interactions. The module’s educational effectiveness was validated with objective measures, such as electroencephalography and mood assessments.

**Objective:**

The authors aimed to extend the module by integrating a radiation visualization system that renders otherwise invisible radiation fields within an immersive VR environment, and to compare students’ learning experiences associated with the module with those associated with conventional hands-on training.

**Methods:**

Twenty students enrolled in a radiological technician training program participated in this study. Students’ learning experiences were evaluated using Likert-scale–based questionnaires, together with qualitative analysis of open-ended responses.

**Results:**

VR-based training was associated with significantly higher ratings for students’ conceptual understanding of radiation behavior, radiation safety awareness, learning satisfaction, and self-assessed competency than conventional training. Conversely, perceived realism and immersion were rated significantly higher following conventional training. No significant differences were observed for operational understanding, technical skills, or system usability.

**Conclusions:**

These findings suggest that the developed VR module offers a safe and repeatable training platform that may support positively perceived learning experiences in radiopharmaceutical administration and radiation safety education.

## Introduction

Virtual reality (VR)–based training is increasingly recognized as a valuable modality in health professions education, offering immersive, safe, and controlled environments for learning complex clinical skills that are difficult to replicate in traditional clinical training settings [1]. Systematic reviews have indicated that VR yields greater engagement, competence, and confidence across diverse medical and radiology training contexts than traditional teaching methods [1,2]. In radiology education, VR has been shown to improve proficiency in patient positioning, equipment handling, and radiographic techniques, with students demonstrating improved perceived competence and satisfaction. Moreover, immersive technologies such as VR and mixed reality (MR) have demonstrated significant improvements in learning outcomes, performance, and engagement in medical and radiation physics education [3].

Beyond general clinical skills training, VR applications are increasingly being explored in radiation safety education. 3D VR simulations have demonstrated an ability to enhance students’ understanding of radiation protection principles and promote active engagement with safety practices, with learners reporting positive perceptions of VR as an educational tool [4]. Comparative studies have further suggested that VR-based training can outperform conventional lecture-based radiation safety training, leading to improved knowledge retention and reduced simulated occupational exposure among health care professionals [5].

Our research group previously developed a VR-based training module for radiopharmaceutical administration focusing on procedural performance and simulated patient interactions. This module was evaluated using physiological and psychological measures, including electroencephalography and mood-state assessments, and yielded promising educational outcomes [6]. Although the previous system included basic visualization features, the representation of radiation fields had limited fidelity and was not fully grounded in clinical measurement data. Therefore, in this study, we developed a specialized radiation visualization system designed to represent radiation distribution and exposure risk with enhanced realism and clinical accuracy. Spatial dose measurements acquired from clinical settings were used to construct a 3D map of radiation fields within the VR environment, enabling the reproduction of radiation distribution patterns that closely approximate real clinical conditions. By integrating clinically measured dose data into an immersive VR space, we aimed to examine students’ learning experiences associated with data-driven, high-fidelity visualization of radiation, including conceptual understanding, radiation-safety awareness, perceived realism and immersion, learning satisfaction, and self-perceived competence, compared with conventional hands-on training.

The primary objective of this study was to systematically evaluate students’ learning experiences associated with perceptual radiation visualization in a clinically grounded VR-based training environment. By integrating 3D radiation field mapping with spatial dose measurements acquired from real clinical settings, a high-fidelity, data-driven simulation platform was developed. Using subjective evaluations and qualitative feedback, the study systematically examined students’ perceptions of their understanding of radiation, radiation safety awareness, and learning experiences associated with the VR environment. Through this approach, we aimed to provide insights into the potential role of perceptual visualization in radiation safety education and the application of experiential learning in radiopharmaceutical administration training.

## Methods

### Study Design

This study adopted a prospective, within-participants, comparative, quasi-experimental, mixed methods design to compare students’ learning experiences associated with a VR-based radiopharmaceutical administration training module incorporating a radiation visualization system (VR-based training) and conventional radiopharmaceutical administration training (conventional training), using both quantitative and qualitative methods.

### Ethical Considerations

This study was conducted in accordance with the Declaration of Helsinki and was approved by the Institutional Ethics Committee of Morinomiya University of Medical Sciences (approval number: 2025‐069). All eligible students were provided with a detailed explanation of the study’s purpose, procedures, and data management processes, and electronic informed consent was obtained via Microsoft Forms prior to enrollment. Participation was entirely voluntary, and students were explicitly informed that participation or nonparticipation would not influence their academic evaluations. All data were anonymized and securely stored to prevent individual identification. Data collection was initiated only after the release of final grades for the relevant academic terms to eliminate any potential influence on student evaluations. Study information sheets, consent forms, and withdrawal forms were distributed in paper format, and consent records were managed using Microsoft Forms.

The study period spanned from April 1, 2025, to January 31, 2026, and volunteer-based measurements were performed between December 1, 2025, and December 23, 2025.

### VR System Development and Visualization Design

The VR-based training system was built on a PC running Windows 10 Pro (64-bit) equipped with an NVIDIA GeForce RTX 2080 graphics card. The hardware configuration consisted of a VIVE Pro Eye head-mounted display (HMD), VIVE Tracker 3.0 devices, and the SteamVR Base Station 2.0 (High Tech Computer Corp) for motion tracking, and Vib+[L] VR gloves (Mollisen) for precise hand interaction. The software environment was constructed using Unity (version 2023.3.7f1; Unity Technologies), supported by the SteamVR Plugin (version 2.7.4; software development kit [SDK] 1.14.15), SteamVR (version 2.2.3), and the Mollisen SDK.

The system configuration is illustrated in Figure 1. The HMD was connected to the PC via a wired connection, while the VR gloves communicated wirelessly. Each glove was equipped with a dedicated tracker for spatial tracking. Using the SteamVR Base Station system, the position and orientation of the HMD and gloves were continuously monitored and transmitted to the VR application, enabling real-time synchronization of user movements within the virtual environment. The VR-based training software was executed on the Unity platform. By wearing an HMD and VR gloves, users could interact with objects in the virtual space. The finger motion data captured from the gloves were integrated using the Mollisen SDK, enabling accurate reproduction of the user’s finger movements within the VR-based training environment.

**Figure 1.**
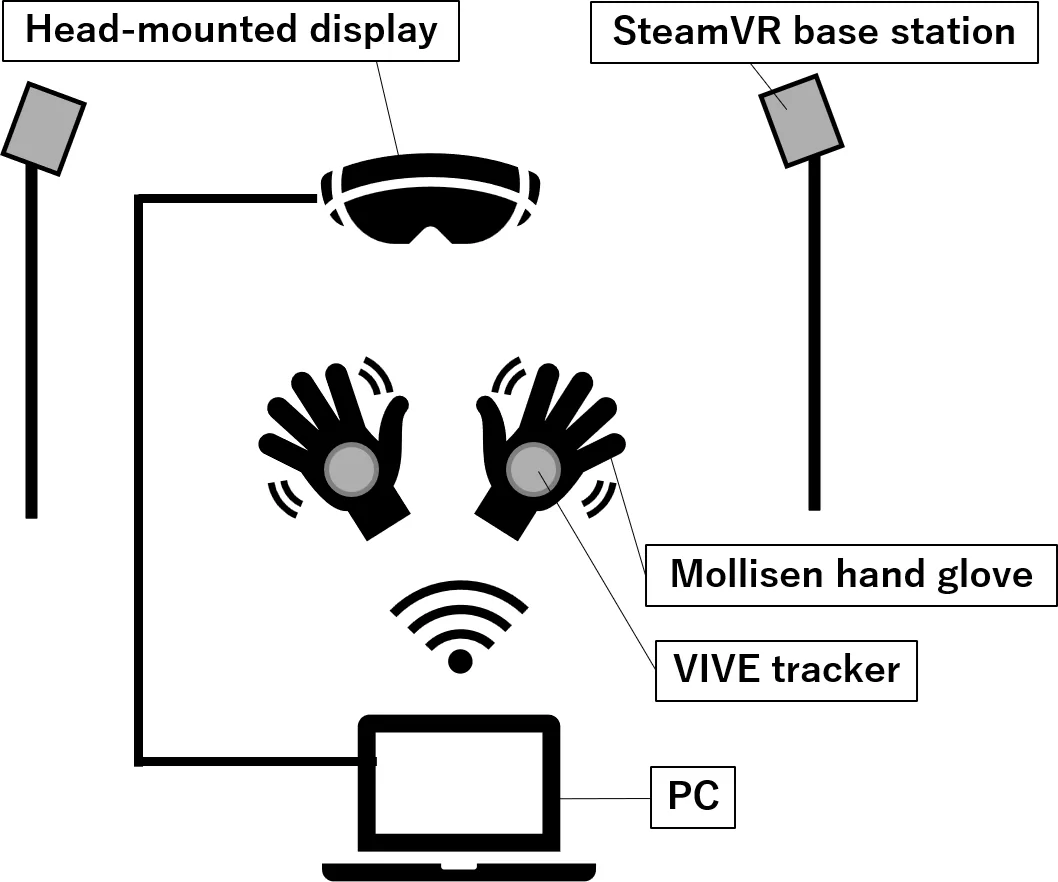
Virtual reality (VR) system configuration diagram.

The developed VR module is presented in Figure 2. Figure 2A shows conventional training, whereas Figure 2B depicts VR-based training using an HMD and dedicated VR gloves. Figure 2C shows learners’ viewpoints within the VR-based training environment. To support the visualization of radiation exposure during training, radiation field visualization data were incorporated into the VR environment. Figure 2D and E present the visualized dose-rate distributions without and with a syringe shield, respectively. These visualizations can be activated or deactivated via interface buttons, allowing learners to directly compare shielding effects during task performance.

**Figure 2.**
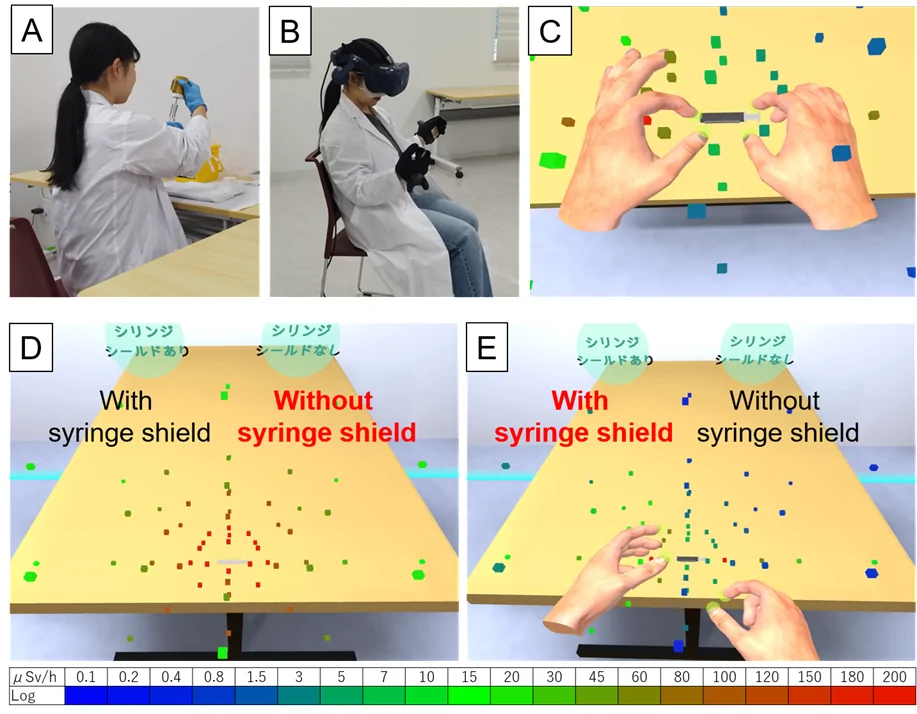
Radiopharmaceutical administration training: conventional and virtual reality (VR)–based methods. (A) Conventional training conducted in a real clinical environment. (B) VR-based training performed using a head-mounted display and VR gloves. (C) First-person perspective of the learner’s hands within the VR-based training environment. (D) Radiation field visualization in the absence of a syringe shield. (E) Radiation field visualization with a syringe shield in place.

Radiation field visualization was constructed from empirical measurements obtained using Technetium-99m (⁹⁹ᵐTc), the radionuclide most commonly used in nuclear medicine, to more closely represent radiation distributions encountered in clinical practice. Radioactivity was measured using a curiemeter (IGC-7F; ALOKA Co, Ltd), and spatial dose rates were measured using a NaI(Tl) scintillation survey meter (NHC6; Fuji Electric Co, Ltd), which was calibrated annually by an accredited external service provider. Dose rates were measured at predefined locations surrounding the vial and syringe (10, 20, 30, and 50 cm at predetermined horizontal and vertical angles), and the mean values from 3 repeated measurements were used to generate the spatial dose distribution. Although the measurements were performed using 68.6 MBq of ⁹⁹ᵐTc, which is lower than activities commonly administered in clinical practice, this activity was selected to minimize radiation exposure during the prolonged measurement procedure and to remain within the operating range of the survey meter. Because the objective was to reproduce the spatial distribution of the radiation field rather than absolute dose rates, the measured dose distribution was normalized and implemented as transparent fixed objects in the VR environment. As shown in Figure 2, the measured data were visualized as a discrete spatial distribution in the VR environment. Dose rate values were presented using a conventional blue-green-red rainbow color scale and were rendered on a logarithmic scale rather than a linear scale to enhance perceptual discrimination of radiation intensity gradients (Multimedia Appendix 1). As the air dose rate from a radiation source decreases with distance according to the inverse-square law [7], variations in the low-dose region become difficult to distinguish when visualized on a linear scale. Therefore, in this study, dose values were color-mapped using a logarithmic scale to maintain perceptual contrast across a wide dynamic range of radiation intensities [8,9].

The color bar indicates the corresponding dose-rate values used in the radiation visualization; detailed color maps are provided in Multimedia Appendix 1.

### Participants and Procedure

A total of 20 third-year undergraduate radiological technology students volunteered to participate in this study (n=10 male and n=10 female participants; mean age 20.9, SD 0.45, range: 20-22 years). All participants had completed the required technical skills and patient communication training before participation in this study. In addition, they had prior experience with both conventional cold-run training and supervised hot-run training involving unsealed radioactive sources during their nuclear medicine practical training approximately 5 months before the study. Therefore, they were not novices with respect to radiopharmaceutical administration procedures.

The study procedure was as follows: first, participants completed conventional training using vials, syringes, and physiological saline as surrogates for unsealed radioactive sources (cold run), which involved no actual radiation exposure. Next, participants wore an HMD and VR gloves to complete the same tasks in the VR-based training environment, which closely simulated real-world clinical conditions and incorporated radiation-field visualization. Each session consisted of brief tasks lasting approximately 5 minutes. Finally, participants completed a web-based questionnaire to report their learning experiences and perceptions.

### Quantitative Evaluation

A quantitative evaluation was conducted using a self-administered questionnaire based on a 5-point Likert scale (1=“strongly disagree,” 2=“disagree,” 3=“neutral,” 4=“agree,” and 5=“strongly agree”) [10]. The questionnaire consisted of 7 primary domains and 35 secondary items, designed to reflect hierarchical learning progression from basic understanding to independent performance and the ability to explain or teach others (Multimedia Appendix 2). The questionnaire was used to assess participants’ self-reported perceptions and learning experiences associated with the training modules.

A questionnaire previously developed and validated by our research group for evaluating learning experiences in nuclear medicine practical training was adapted for the present study [11]. The original instrument underwent expert review by 3 faculty members specializing in nuclear medicine education and radiological technology, followed by pilot testing involving 10 students and 6 faculty members who were not involved in the questionnaire’s development. The validation study demonstrated excellent internal consistency, as assessed using Cronbach α of 0.91 [11,12], with domain-specific Cronbach α values ranging from 0.68 to 0.90. Preliminary construct validity was supported through exploratory factor analysis using the Kaiser-Meyer-Olkin (KMO) measure [13] and Bartlett test of sphericity [14], which demonstrated acceptable sampling adequacy (KMO=0.85 and 0.81 for cold- and hot-run datasets, respectively; Bartlett test of sphericity, *P*<.001 for both datasets [11]). The extracted factor structure was generally consistent with the intended educational domains, providing preliminary support for the construct validity of the instrument [11]. For this study, the questionnaire structure was retained and modified to include items related to radiation visualization, VR usability, and learning experiences associated with the VR-based training environment.

The 7 primary domains were as follows: (A) radiation understanding and visualization, (B) operational understanding and technical skills (vial and shielding manipulations), (C) perceived realism and immersion, (D) medical safety awareness and radiation protection, (E) system evaluation (usability and performance), (F) learning satisfaction, and (G) self-evaluation.

The radiation understanding and visualization domain (A) assessed intuitive comprehension of spatial radiation distribution, understanding of shielding effects (with and without syringe shields), perception of radiation intensity and attenuation through color mapping, improvement in safety awareness, and the ability to communicate spatial dose distribution concepts to peers.

The operational understanding and technical skills domain (B) evaluated the comprehension of equipment structure, appropriate use of syringe shields, perceived realism of operational sensations (eg, weight and spatial positioning), conscious integration of radiation protection principles during task execution, and confidence in explaining these procedures to others.

The perceived realism and immersion domain (C) investigated perceived realism, correspondence with real clinical working environments, naturalness of hand and vial manipulation, the ability to maintain concentration, and the capacity to articulate task-related tension. Item C2, which assessed a sense of presence similar to a real working environment, was interpreted as an indicator of perceived realism.

The medical safety awareness and radiation protection domain (D) examined understanding of safety considerations when handling unsealed radioactive sources; awareness of dose-reduction strategies; recognition of the principles of distance, shielding, and time; the perceived importance of radiation protection; and confidence in instructing others.

The system evaluation domain (E) evaluated interface clarity, operational smoothness, absence of operational discomfort, perceived value for repeated practice, and perceived educational significance for integration into future training curricula.

The learning satisfaction domain (F) assessed perceived acquisition of new knowledge, a deeper understanding of radiation and safety management, satisfaction with the training experience, perceived applicability to future professional practice, and willingness to continue using similar educational tools.

The self-evaluation domain (G) assessed confidence in newly acquired knowledge and skills, perceived ability to independently perform comparable procedures, confidence in executing procedures safely in future clinical practice, heightened awareness of radiation handling, and willingness to recommend this learning approach to peers.

Primary outcome scores were estimated as the mean of the 5 corresponding secondary items within each domain. Secondary outcome items were analyzed separately to identify specific aspects of participants’ learning experiences. The mean scores were compared between conventional (cold-run) training and VR-based training.

Statistical analyses were conducted using the Wilcoxon signed-rank test because the questionnaire responses were measured on a 5-point Likert scale. Effect sizes (*r*) were also calculated to evaluate the magnitude of the observed differences. Statistical significance was initially defined as *P*<.05. To account for multiple comparisons, Bonferroni correction was applied separately to the primary outcomes (7 domains; adjusted α=.0071) and secondary outcomes (35 items; adjusted α=.0014). All quantitative analyses were performed using Microsoft Excel.

### Qualitative Evaluation

In this study, open-ended responses corresponding to H1 to H3 were analyzed using a qualitative descriptive approach based on Kawakita Jiro method (KJ method), a systematic technique for organizing and interpreting qualitative data [15], supplemented by established qualitative analysis frameworks [16-18]. To ensure analytical neutrality, the analyses were conducted by 2 researchers who were not involved in the educational instruction or VR-based training.

The open-ended survey items analyzed were as follows:

- H1: Please describe any impressions or insights gained from the radiation field visualization experience.
- H2: Please describe any perceived differences between performing tasks in a real-world environment and in a VR environment.
- H3: Please describe any desired improvements or additional functions you would recommend for the system.

The analysis commenced with repeated readings of all responses to familiarize the researchers with the data and to identify meaningful statements related to radiation field visualization experiences, operational differences between conventional and VR-based training, and suggestions for system improvement. These statements were divided into discrete meaning units, grouped according to content similarity, and assigned descriptive labels. The 2 researchers independently reviewed and coded the responses before collaboratively comparing and refining the resulting codes, categories, and themes through discussion until consensus was reached. Following the principles of the KJ method, related groups were systematically compared and integrated into higher-order categories, from which overarching themes reflecting learners’ subjective perceptions were extracted. This consensus-based process was used to enhance the credibility and consistency of the qualitative analysis.

This approach enabled the systematic extraction of learners’ subjective experiences and insights into the educational impact that could not be adequately quantified using a Likert-scale–based quantitative approach [10].

## Results

### Quantitative Evaluation

Quantitative analyses were performed to examine differences in participants’ self-reported learning experiences and perceptions between conventional and VR-based training. The 7 primary evaluation categories described in the “Methods” section were assessed using a Likert scale.

#### Primary Outcomes

The primary outcomes of this study are presented in Table 1. All 20 participants completed both conventional and VR-based training sessions and submitted questionnaires for each type of training. The 7 primary outcome domains demonstrated good internal consistency, with Cronbach α values of 0.822 and 0.850 for the conventional and VR-based training questionnaires, respectively.

**Table 1.** Comparison of primary outcomes between conventional training and virtual reality (VR)–based training.

Primary outcomes	Conventional training, mean (SD)	VR-based training, mean (SD)	Wilcoxon signed-rank test	*P* value	Effect size (*r*)	Significance (α=.0071)
(A) Radiation understanding and visualization experience	1.85 (0.45)	4.70 (0.25)	0	<.001	0.878	—^a^
(B) Operational understanding and technical skills (vial and shielding manipulation)	3.60 (0.52)	3.55 (0.66)	80.5	.83	0.049	n.s.^b^
(C) Perceived realism and immersion	4.44 (0.52)	3.48 (0.70)	9	<.001	0.774	—^a^
(D) Medical safety awareness and radiation protection	3.07 (0.57)	4.35 (0.46)	0	<.001	0.856	—^a^
(E) System evaluation (usability and performance)	4.24 (0.65)	4.05 (0.65)	54	.29	0.239	n.s.
(F) Learning satisfaction	3.88 (0.47)	4.52 (0.46)	0	<.001	0.835	—^a^
(G) Self-evaluation	3.81 (0.64)	4.48 (0.44)	0	<.001	0.811	—^a^

a Indicates statistical significance.

b n.s.: nonsignificant.

For the (A) radiation understanding and visualization domain, the mean score for conventional training was 1.85  (SD 0.45), whereas VR-based training yielded a significantly higher mean score of 4.70 (SD 0.25). The (B) operational understanding and technical skills (vial and shielding manipulation) domain showed comparable results across both training modalities, with mean scores of 3.60 (SD 0.52) for conventional training and 3.55 (SD 0.66) for VR-based training. In the (C) perceived realism and immersion domain, conventional training was rated higher (mean 4.44, SD 0.52) than VR-based training (mean 3.48,  SD 0.70).

The (D) medical safety awareness and radiation protection domain yielded mean scores of 3.07 (SD 0.57) for conventional training and 4.35 (SD 0.46) for VR-based training. The (E) system usability and performance domain demonstrated comparable scores for conventional training and VR-based training (mean 4.24, SD 0.65 vs mean 4.05, SD 0.65). The (F) learning satisfaction domain yielded mean scores of 3.88 (SD 0.47) for conventional training and 4.52 (SD 0.46) for VR-based training, while (G) self-evaluation domain yielded mean scores of 3.81 (SD 0.64) for conventional training and 4.48 (SD 0.44) for VR-based training, with higher ratings reported for VR-based training in both domains.

Wilcoxon signed-rank tests revealed that participants reported significantly higher ratings for categories (A), (D), (F), and (G) following VR-based training than following conventional training, whereas higher ratings were reported for conventional training in category (C). No significant differences were observed in categories (B) and (E). The significant differences were generally associated with large effect sizes.

#### Secondary Outcomes

The secondary outcomes for each evaluation category are presented in Table 2, which shows the mean score (SD) for each item in both conventional and VR-based training. All 20 participants completed questionnaires for both training modalities. The questionnaire revised for this study, including additional items related to radiation visualization and VR-based training, demonstrated good internal consistency, with Cronbach α values of 0.893 and 0.914 for the conventional and VR-based training questionnaires, respectively.

**Table 2.** Comparison of secondary outcomes between conventional training and virtual reality (VR)–based training.

Secondary outcomes	Conventional training, mean (SD)	VR-based training, mean (SD)	Wilcoxon signed-rank test	*P* value	Effect size (*r*)	Significance (α=.0014)
A1	1.55 (0.83)	4.70 (0.47)	0	<.001	0.890	—^a^
A2	1.80 (0.77)	4.70 (0.47)	0	<.001	0.893	—^a^
A3	1.20 (0.52)	5.00 (0)	0	<.001	0.946	—^a^
A4	2.75 (0.72)	4.70 (0.47)	0	<.001	0.888	—^a^
A5	1.95 (0.76)	4.40 (0.50)	0	<.001	0.886	—^a^
B1	3.80 (1.06)	3.55 (0.94)	63.0	.51	0.146	n.s.^b^
B2	3.25 (0.72)	3.90 (0.97)	37.5	.06	0.427	n.s.
B3	4.60 (0.60)	2.40 (1.23)	0	<.001	0.794	—^a^
B4	3.05 (0.83)	4.00 (0.86)	5.5	.001324^c^	0.718	—^a^
B5	3.30 (0.98)	3.90 (0.64)	0	.002282^c^	0.682	n.s.
C1	4.20 (1.01)	3.45 (0.83)	38.0	.04	0.473	n.s.
C2	4.75 (0.44)	3.20 (0.95)	0	<.001	0.821	—^a^
C3	4.85 (0.37)	2.85 (1.04)	0	<.001	0.822	—^a^
C4	4.40 (0.82)	4.10 (0.97)	9	.19	0.289	n.s.
C5	4.00 (0.79)	3.80 (1.01)	41.5	.48	0.160	n.s.
D1	3.25 (0.64)	4.40 (0.68)	5.5	<.001	0.774	—^a^
D2	3.15 (0.67)	4.30 (0.80)	0	<.001	0.783	—^a^
D3	2.95 (0.76)	4.55 (0.76)	0	<.001	0.800	—^a^
D4	3.05 (0.83)	4.75 (0.44)	0	<.001	0.837	—^a^
D5	2.95 (1.00)	3.75 (0.97)	0	.001491^c^	0.710	n.s.
E1	3.75 (1.37)	4.05 (0.94)	29.5	.45	0.170	n.s.
E2	4.45 (0.76)	4.10 (0.91)	23.5	.21	0.282	n.s.
E3	4.50 (0.76)	3.80 (0.89)	23.0	.03	0.487	n.s.
E4	4.20 (0.89)	4.05 (1.10)	50.5	.58	0.122	n.s.
E5	4.30 (0.98)	4.25 (0.72)	25.0	.79	0.058	n.s.
F1	3.25 (0.91)	4.70 (0.47)	0	<.001	0.826	—^a^
F2	3.15 (0.75)	4.70 (0.47)	0	<.001	0.850	—^a^
F3	3.90 (0.72)	4.35 (0.59)	9	.05	0.439	n.s.
F4	4.60 (0.60)	4.35 (0.81)	10.5	.27	0.246	n.s.
F5	4.50 (0.69)	4.50 (0.69)	27.5	>.99	0	n.s.
G1	3.20 (1.20)	4.60 (0.60)	0	<.001	0.804	—^a^
G2	3.90 (1.12)	3.85 (1.04)	20.0	.76	0.068	n.s.
G3	4.10 (0.72)	4.65 (0.49)	0	.008	0.589	n.s.
G4	3.55 (0.83)	4.75 (0.44)	6	<.001	0.799	—^a^
G5	4.30 (0.66)	4.55 (0.51)	20.0	.22	0.275	n.s.

a Indicates statistical significance.

b n.s.: nonsignificant.

c *P* values close to the Bonferroni-adjusted significance threshold (α=.0014), requiring additional precision to distinguish statistically significant from nonsignificant results*. *B4 is statistically significant, whereas B5 and D5 are nonsignificant.

For (A) radiation understanding and visualization domain, all 5 subitems (A1-A5) demonstrated markedly higher scores with VR-based training than with conventional training. For example, A1 (“intuitive understanding of the spatial extent and distribution of radiation”) had a mean score of 1.55 (SD 0.83) for conventional training and 4.70 (SD 0.47) for VR-based training, and A3 (“visual perception of radiation intensity and attenuation through color changes”) increased from 1.20 (SD 0.52) to 5.00 (SD 0).

For (B) operational understanding and technical skills (vial and shielding manipulation) domain, results varied across items. B3 (“sufficiently experienced realistic operational sensations, such as weight and spatial positioning”) was higher in conventional training (mean 4.60, SD 0.60) than in VR-based training (mean 2.40, SD 1.23), whereas B4 (“performed tasks while consciously applying radiation protection principles”) increased from a mean score of 3.05 (SD 0.83) in conventional training to 4.00 (SD 0.86) in VR-based training. The remaining items showed no substantial differences between the 2 training modalities.

For (C) perceived realism and immersion domain, conventional training scored higher on some items, such as C2 (“sense of presence similar to an actual working environment”; mean 4.75, SD 0.44 vs mean 3.20, SD 0.95); however, the remaining items were comparable between the 2 methods.

For (D) safety awareness and radiation protection domain, VR-based training yielded higher scores for most items. For instance, the mean score of D3 (“recognition of the importance of distance, shielding, and time principles through the experience”) increased from 2.95 (SD 0.76) in conventional training to 4.55 (SD 0.76) in VR-based training; however, similar patterns were observed for D1, D2, and D4.

For (E) system usability and performance domain, similar scores were observed across all items; however, no statistically significant differences were observed between conventional and VR-based training for any item in this domain.

For (F) learning satisfaction domain, “higher ratings were observed for VR-based training in” F1 (mean 3.25, SD 0.91 vs mean 4.70, SD 0.47) and F2 (mean 3.15, SD 0.75 vs mean 4.70, SD 0.47); however, the remaining items were comparable between the 2 methods.

Finally, in (G) self-evaluation domain, VR-based training yielded higher scores for G1 (mean 3.20, SD 1.20 vs mean 4.60, SD 0.60) and G4 (mean 3.55, SD 0.83] vs mean 4.75, SD 0.44) than conventional training. The remaining items did not differ substantially.

Overall, participants reported higher ratings for VR-based training primarily in domains related to radiation understanding and visualization, medical safety awareness and radiation protection, learning satisfaction, and self-evaluation, whereas conventional training received higher ratings for selected items related to physical and operational realism.

### Qualitative Findings: Comparison Between Real and VR Environments

#### Overview

Open-ended responses from 20 participants were analyzed using a qualitative descriptive approach based on the KJ method [15]. The frequencies presented below are intended to provide a descriptive summary of the occurrence of participant comments and should not be interpreted as quantitative outcome measures. A summary of the identified qualitative themes and the number of participants contributing to each theme is presented in Table 3. Following this methodology, the following five key themes were identified from the participants’ responses:

1. Radiation field visualization in VR: Participants described how the radiation distribution, invisible during conventional training, became perceptible within the VR-based training environment.
2. Radiation safety awareness in VR: Participants reported heightened attention to radiation protection while operating in the VR-based training environment.
3. Operational differences between conventional and VR-based training: Participants compared tactile sensations, weight perception, and procedural manipulation across both training modalities.
4. Practical skill acquisition in conventional training: Participants emphasized that hands-on practice in the conventional training environment remained important for procedural skill development.
5. VR-based training usability and suggestions for improvement: Participants reported operational difficulties and proposed recommendations for enhancing the VR-based training system.

**Table 3.** Summary of qualitative themes identified from participants’ open-ended responses (N=20).

Theme	Participants^a^, n
Radiation field visualization in VR^b^	20
Radiation safety awareness in VR	15
Operational differences between conventional and VR-based training	20
Practical skill acquisition in conventional training	8
VR-based training usability and suggestions for improvement	10

a “Participants” indicates the number of participants contributing to each theme. Individual participants could contribute to more than one theme; therefore, the totals exceed 20.

b VR: virtual reality.

The following subsections summarize participants’ responses according to these 5 themes.

#### Radiation Field Visualization in VR-Based Training

All participants (20/20) reported that radiation, which was not directly observable during conventional training, became perceptible in the VR-based training environment. Representative statements included the following:

*Radiation could not be observed during conventional training; however, in the VR-based training environment, the radiation field became visible.*

*Visualizing this field allowed me to identify areas of high exposure risk.*

*Observing the depth and spread of the radiation made the associated risks easier to understand.*

#### Radiation Safety Awareness in VR

Fifteen participants stated that VR-based training heightened their radiation safety awareness. Representative statements included the following:

*In the VR-based training environment, I was able to perform the procedure while simultaneously considering radiation exposure.*

*Visualizing the radiation field heightened my attention to safety management.*

*I became more conscious of shielding and maintaining distance.*

The participants reported that during conventional training, attention was primarily directed toward procedural execution, whereas the VR-based training environment allowed simultaneous consideration of radiation exposure and safety measures.

#### Operability Differences Between Conventional and VR Environments

All participants (20/20) reported differences in operability between the conventional and VR-based training environments. Common observations included the following:

- The presence of tactile feedback and weight perception in the conventional training environment.
- Absence of haptic feedback in the VR-based training environment.
- Challenges in accurately judging spatial distance within the VR-based training environment.
- Difficulties in manipulating interface controls during VR-based training.

Representative comments included the following:

*In the conventional training environment, I could feel the weight of the syringe.*

*In the VR-based training environment, it was difficult to perceive the distance.*

*Operating the interface buttons in VR-based training was more challenging than handling real objects.*

Participants frequently compared tactile feedback, weight perception, spatial distance perception, and interface operability between the conventional and VR-based training environments.

#### Practical Skill Acquisition in Conventional Training

Eight participants emphasized that procedural skills could only be reliably developed in a conventional training environment. Representative statements included the following:

*In the conventional training environment, I was able to practice actual manipulations.*

*Handling real objects deepened my understanding of their physical structure and weight.*

*The conventional training environment was superior for procedural skill development.*

Participants emphasized the importance of hands-on practice using real objects for procedural skill acquisition.

#### VR-Based Training Usability and Improvement Suggestions

Ten participants reported usability limitations in the VR-based training and proposed the following targeted system improvements:

- Difficulty in accurately judging spatial distances within the VR-based training environment.
- Visual focus misalignment experienced during VR-based training.
- Operational difficulties with VR interface controls.
- Requests for more realistic procedural tasks.

Representative comments included the following:

*Visual focus did not align accurately within the VR-based training environment, and determining the distance to interface buttons was challenging. It would be better if actual injection procedures could be performed during the VR-based training.*

Participants frequently reported technical limitations related to spatial perception, visual alignment, interface operation, and procedural realism in the VR-based training environment.

## Discussion

### Principal Findings

Participants reported higher ratings for radiation field understanding and visualization, radiation safety awareness, learning satisfaction, and self-evaluation following VR-based training than following conventional training. The improvements in radiation field understanding and visualization were expected because visualization of otherwise invisible radiation was a core design feature of the VR system and should therefore be interpreted as confirmation of the intended system functionality rather than independent evidence of educational superiority. By contrast, no significant differences were observed in operational understanding, technical skills, or system usability. Notably, perceived realism and immersion scores were significantly higher in the conventional training environment than in the VR-based training. Because all participants had already completed both conventional cold-run training and supervised hot-run training approximately 5 months before this study, these findings should be interpreted as reflecting perceptions formed after prior practical experience rather than those of novice learners.

These quantitative findings were supported by qualitative analysis. Participants emphasized the value of radiation field visualization in VR-based training, whereas the higher ratings for realism-related items were considered to reflect inherent characteristics of the respective training environments. The qualitative findings also suggested that the lower realism and immersion ratings for the VR-based training were primarily attributable to limitations in tactile feedback and object manipulation rather than to the educational content itself. Future advances in VR hardware, including improvements in haptic feedback, hand tracking, and display technologies, may further enhance realism and immersion in VR-based training. Collectively, these results suggest that VR-based training may support positive perceived learning experiences related to cognitive and conceptual learning domains, whereas conventional training retains advantages in experiential immersion and psychomotor realism. Beyond the expected benefit of radiation field visualization, the observed improvements in radiation safety awareness, learning satisfaction, and self-evaluation suggest that the educational value of VR-based training extends beyond visualization alone.

### Improvement in Radiation Visualization and Safety Awareness

The most substantial effect of VR-based training was observed in radiation field understanding and visualization (*P*<.001). Because radiation is inherently invisible, conventional training based on static diagrams and theoretical explanations may limit learners’ intuitive understanding of spatial dose distribution and shielding effects.

Previous research has highlighted the limitations of lecture-based radiation safety education and the need for practical, perceptually enriched training strategies [19]. Previous VR studies have used interactive radiation field visualization to support learners’ understanding of spatial dose distributions, shielding, exposure patterns, and protective positioning [20,21]. The current findings are consistent with previous reports showing that VR-based training was associated with higher ratings for radiation field visualization and radiation safety awareness. These findings suggest that immersive radiation visualization may help bridge the gap between abstract radiation concepts and practical radiation safety education.

### Learning Satisfaction and Self-Evaluation

Learning satisfaction (*P*<.001) and self-evaluation (*P*<.001) were significantly higher following VR-based training. These gains are likely attributable to the interactive nature, immediate visual feedback, and immersive simulation afforded by the VR-based training environment. However, these findings should be interpreted with caution because the novelty of the immersive VR environment and the inability to blind participants may have contributed to higher satisfaction and self-evaluation ratings.

Previous meta-analyses in health care education have demonstrated that immersive VR-based training yielded greater improvements in learner satisfaction than conventional training [22]. Immersive environments foster emotional engagement and perceived educational value, positively influencing the motivational dimensions of learning [23]. VR has the potential to promote engagement and active participation, which are important factors influencing educational satisfaction [24]. In our previous investigation of VR-based training for radiopharmaceutical administration, higher levels of a sense of presence during VR-based training were associated with improved perceived educational outcomes [6]. The qualitative responses in this study similarly indicated that participants perceived VR-based training as an educationally meaningful tool. Collectively, these findings suggest that VR-based training may support both the conceptual and motivational dimensions of participants’ perceived learning experiences.

### Operability and Technical Skills

No significant differences were observed in operational understanding or technical skills (*P*=.83), indicating that the current VR-based training implementation did not outperform conventional training in psychomotor skill acquisition. Although conceptual comprehension improved, procedural performance remained comparable across both training modalities.

Qualitative analysis revealed frequent references to operability differences and the importance of tactile feedback in conventional training environments. Participants consistently emphasized that physical resistance, fine motor control, and authentic material manipulation were critical contributors to procedural mastery. Likewise, limitations in haptic fidelity and usability have been recognized as barriers to effective psychomotor skill transfer in VR-based training [25,26], which is consistent with the qualitative findings of the present study.

Technical constraints related to finger tracking and standardized VR glove sizing may have reduced fine motor precision and perceived operability, particularly when the glove dimensions did not adequately match participants’ hand sizes. These factors may partly explain why VR-based training did not outperform conventional training in perceived psychomotor skills.

These findings suggest that limitations in haptic realism, ergonomic adaptability, and interface customization may constrain perceived technical skill development. Adaptive haptic systems and customizable interfaces may help address these limitations.

### Perceived Realism and Immersion

Notably, ratings for perceived realism and immersion were significantly higher (*P*<.001) in conventional training than in VR-based training. Participants may have rated the conventional training environment as more realistic and immersive because of multisensory integration, tactile feedback, and environmental complexity.

In this study, the marked differences in object weight (3.86 g and 13.3 g without shielding vs 114 g and 602 g with shielding) likely contributed to this perceptual discrepancy. These differences provided proprioceptive, kinesthetic, and tactile cues that were absent in the current VR-based training environment, where virtual objects had no physical weight. Such a fundamental incongruence between actual force feedback and the visual representation may reduce the perceived physical realism and immersion in VR-based training, particularly during fine motor tasks that require accurate weight discrimination and resistance perception.

Previous research has demonstrated that incorporating haptic feedback into VR-based training enhances realism and learner confidence in procedural tasks [27,28]. Consistent with these previous findings, participants in the present study reported lower ratings for perceived realism and immersion in the absence of realistic weight perception and tactile feedback. These findings suggest that perceived immersion may depend not only on visual fidelity but also on congruent multisensory inputs.

Emerging MR approaches may help address this sensory incongruence by allowing interaction with real objects while overlaying digital guidance, thereby preserving the authentic physical properties of weight and resistance [29]. Moreover, advances in tactile technologies for VR-based training in radiation education suggest that integrating sensor-based or vibrotactile feedback into VR glove systems could further strengthen perceived realism, immersion, fine-motor realism, and overall perceived operability [30]. Collectively, these findings suggest that the 2 training modalities engage different experiential and sensorimotor mechanisms. Integrating MR and advanced haptic systems may therefore combine the visual advantages of VR-based training with the tactile authenticity of conventional training.

### Educational Implications

The findings indicate that VR-based training may be particularly well-suited to undergraduate radiological education, where students are still developing core procedural competencies and foundational radiation protection awareness. VR-based training transforms abstract and imperceptible radiation phenomena into visually and spatially interpretable representations, which may facilitate conceptual understanding of radiation and enhance perceived learning experiences. Accordingly, these findings are most applicable to undergraduate radiological technology students who have already acquired fundamental procedural skills through conventional practical training.

Conventional training remains important for tactile engagement and realistic procedural experience but does not provide direct visualization of spatial radiation distributions. These findings support a complementary framework in which VR-based training reinforces conceptual understanding and radiation protection awareness alongside conventional practical training.

### Limitations

This study has some limitations that should be considered when interpreting the findings.

First, all participants completed the conventional training before the VR-based training. Therefore, potential order, practice, fatigue, and recency effects could not be separated from the effects of the training modality. Although all participants had previously completed both conventional cold-run training and supervised hot-run training and therefore were not novices, the influence of the fixed-order design cannot be excluded. Consequently, the observed differences should be interpreted as associations rather than evidence of causal educational effects. Future studies using randomized or counterbalanced study designs are warranted to better isolate the educational effects of VR-based training. Furthermore, this study evaluated participants’ perceived learning experiences using self-reported questionnaires and qualitative feedback and did not include objective assessments of knowledge acquisition or procedural performance. Future studies incorporating objective educational outcome measures, such as knowledge tests and performance-based assessments, are warranted to provide a more comprehensive evaluation of the educational effectiveness of VR-based training.

Second, this study was conducted at a single institution with a relatively small sample size consisting of undergraduate students, which may restrict the generalizability to broader educational contexts or to experienced health care professionals. As the participants were students in the early stages of training, the results primarily reflect educational effects at a foundational developmental level and cannot be extrapolated to established clinical or professional competence.

Third, the study relied exclusively on self-reported perceptual measures and lacked objective assessments of operational or procedural skills, such as objective structured clinical examination–based evaluations or performance-based metrics. Therefore, conclusions regarding operational skills or procedural competence should be interpreted cautiously.

Fourth, although the questionnaire was adapted from a previously validated instrument, several items were modified to address radiation visualization and VR-based training experiences. Therefore, the psychometric properties of the modified questionnaire should be further examined in future studies.

Fifth, participants were recruited on a voluntary basis, which may have introduced selection bias toward learners with a greater interest in educational technologies.

Sixth, participants completed the familiar conventional training before experiencing the novel VR-based training environment. Therefore, the higher ratings for learning satisfaction and self-evaluations may have been influenced not only by the characteristics of the VR-based training itself but also by novelty effects and demand characteristics. Because blinding was not feasible in this educational intervention, these potential influences cannot be excluded.

Seventh, only immediate posttraining outcomes were assessed; long-term knowledge retention and transfer to authentic clinical practice remain entirely unexamined. The absence of advanced haptic feedback in the VR-based training system, as well as potential variability in VR glove fit, particularly for students whose hand size did not adequately match the glove dimensions, may also have affected the perceived operability, realism, and immersion during VR-based training. Although the VR system was calibrated using dedicated sensors before each training session, and no obvious misalignment between the visualized objects and users’ hand movements was perceived during operation with the HMD and VR gloves, the spatial accuracy of the visualization was not quantitatively evaluated.

Furthermore, because this study included only undergraduate radiological technology students from a single institution who had already completed conventional practical training, the findings may not be generalizable to novice learners, experienced health care professionals, or other educational settings. Future investigations incorporating objective skill assessment, longitudinal follow-up to evaluate knowledge retention, quantitative validation of spatial registration and visualization accuracy, enhanced haptic interfaces and MR technologies in VR-based training, and multicenter validation across different learner populations would strengthen the evidence base for the systematic integration of VR-based training into radiological education.

### Conclusions

This study found that participants reported significantly higher ratings for radiation visualization, radiation protection awareness, learner satisfaction, and self-evaluation following VR-based training, as evidenced by both quantitative and qualitative findings. These findings suggest that VR-based training may provide positive perceived learning experiences related to understanding invisible radiation phenomena and radiation protection awareness. Conversely, conventional training was associated with higher ratings for experiential immersion and realistic operational sensation. Rather than functioning as a substitute for established training methods, VR-based training should be strategically positioned as a complementary educational modality that supports positive perceived learning experiences related to conceptual understanding of radiation and radiation protection awareness.

## Supplementary material

10.2196/104397 Multimedia Appendix 1 Color maps of spatial dose rate distributions.

10.2196/104397 Multimedia Appendix 2 Questionnaire domains and items used for quantitative and qualitative evaluation.

## Abbreviations

HMD: head-mounted display
KJ method: Kawakita Jiro method
KMO: Kaiser-Meyer-Olkin
MR: mixed reality
SDK: software development kit
VR: virtual reality
⁹⁹ᵐTc: Technetium-99m
