# Supplementary material for: Development and Educational Evaluation of a Radiation Visualization System in a Virtual Reality Training Module for Radiopharmaceutical Administration: Mixed Methods Study of Students’ Learning Experiences
Source: JMIR Med Educ. 2026 Sep 30;12:e104397. doi: 10.2196/104397 (PMC13625173; doi:10.2196/104397)
Supplement: Supplementary file 1 [file mededu-v12-e104397-s001.docx]

***Supplementary Material***

**Figure S1.**

**Figure S1. Color maps of spatial dose rate distributions.**

Spatial dose rate distributions are shown under conditions with and without a syringe shield. Measurements were performed using 68.6 MBq of ^99m^Tc. Dose rate values represent the mean of three measurements and were corrected for radioactive decay. Both linear and logarithmic color scales are used to highlight differences in the visual discrimination of radiation intensity.
