# Supplementary material for: Development and Educational Evaluation of a Radiation Visualization System in a Virtual Reality Training Module for Radiopharmaceutical Administration: Mixed Methods Study of Students’ Learning Experiences
Source: JMIR Med Educ. 2026 Sep 30;12:e104397. doi: 10.2196/104397 (PMC13625173; doi:10.2196/104397)
Supplement: Supplementary file 2 [file mededu-v12-e104397-s002.docx]

Table S1. Questionnaire domains and items used for quantitative and qualitative evaluation

| **Primary outcomes** | **Secondary outcomes** | 1 =  strongly disagree | 2 =  disagree | 3 =  neutral | 4 =  agree | 5 =  strongly agree | |
| --- | --- | --- | --- | --- | --- | --- | --- |
| **(A) Radiation understanding and visualization experience** | A1. Intuitively understood the spatial extent and distribution of radiation | □ | □ | □ | □ | □ | |
|  | A2. Understood the shielding effect of radiation (with and without a lead shield) | □ | □ | □ | □ | □ | |
|  | A3. Visually perceived radiation intensity and attenuation through color changes | □ | □ | □ | □ | □ | |
|  | A4. Felt that the experience improved awareness of radiation safety | □ | □ | □ | □ | □ | |
|  | A5. Able to explain the spatial distribution of radiation to others | □ | □ | □ | □ | □ | |
| **(B) Operational understanding and technical skills (vial and shielding manipulation)** | B1. Understood the structure of the equipment and materials | □ | □ | □ | □ | □ | |
|  | B2. Understood the role and proper use of the lead shield | □ | □ | □ | □ | □ | |
|  | B3. Sufficiently experienced realistic operational sensations (e.g., weight and spatial positioning) | □ | □ | □ | □ | □ | |
|  | B4. Performed tasks while consciously applying radiation protection principles | □ | □ | □ | □ | □ | |
|  | B5. Confident in explaining the above procedures to others | □ | □ | □ | □ | □ | |
| **(C) Perceived realism and immersion** | C1. Felt realistic and easy to understand | □ | □ | □ | □ | □ | |
|  | C2. Felt a sense of presence similar to an actual working environment | □ | □ | □ | □ | □ | |
|  | C3. Hand and vial manipulation felt natural | □ | □ | □ | □ | □ | |
|  | C4. Maintained concentration and engaged effectively in the task | □ | □ | □ | □ | □ | |
|  | C5. Able to explain the sense of tension experienced during the task | □ | □ | □ | □ | □ | |
| **(D) Medical safety awareness and radiation protection** | D1. Understood safety considerations when handling unsealed radioactive sources | □ | □ | □ | □ | □ | |
|  | D2. Became aware of practical methods to reduce radiation exposure | □ | □ | □ | □ | □ | |
|  | D3. Recognized the importance of distance, shielding, and time principles through the experience | □ | □ | □ | □ | □ | |
|  | D4. Reaffirmed the importance of radiation protection | □ | □ | □ | □ | □ | |
|  | D5. Confident in instructing others regarding the above safety principles | □ | □ | □ | □ | □ | |
| **(E) System evaluation (usability and performance)** | E1. Found the operational interface easy to understand | □ | □ | □ | □ | □ | |
|  | E2. Felt that the system operated smoothly without causing stress | □ | □ | □ | □ | □ | |
|  | E3. Did not experience discomfort with the display or operation | □ | □ | □ | □ | □ | |
|  | E4. Considered the system valuable for repeated practice | □ | □ | □ | □ | □ | |
|  | E5. Felt that the system has educational significance for future implementation | □ | □ | □ | □ | □ | |
| **(F) Learning satisfaction** | F1. Gained new knowledge through the experience | □ | □ | □ | □ | □ | |
|  | F2. Felt that understanding of radiation and safety management deepened | □ | □ | □ | □ | □ | |
|  | F3. Satisfied with the training content and operation | □ | □ | □ | □ | □ |  |
|  | F4. Felt that the training will be useful in future professional practice | □ | □ | □ | □ | □ |  |
|  | F5. Would like to continue using similar educational tools | □ | □ | □ | □ | □ |  |
| **(G) Self-evaluation** | G1. Felt confident in the knowledge and skills gained through this experience | □ | □ | □ | □ | □ |  |
|  | G2. Felt confident about performing similar procedures independently | □ | □ | □ | □ | □ |  |
|  | G3. Felt more confident about performing the procedure safely in the future | □ | □ | □ | □ | □ |  |
|  | G4. Felt that awareness of radiation handling increased compared with before | □ | □ | □ | □ | □ |  |
|  | G5. Would recommend this learning method to other students | □ | □ | □ | □ | □ |  |
